# Remarkable Case in Obstetrics

**Published:** 1877-03

**Authors:** 


Dr. A. H. Beaton (*The Canada Lancet*) reports the following remarkable case in obstetrics: He was called to a woman in September, 1873, whom, after a somewhat tedious labor, he delivered with the forceps. The child was born with a tumor, nearly as large as its head, in the umbilical cord, about two inches from the abdomen. Otherwise the cord was normal as to length and size. On one side of the tumor was a patch of skin two inches in diameter, of the same color and appearance as the body of the child, the rest of it being membrane resembling the cord, and, indeed, being a portion of it. At first he was at a loss to know what to do, but concluded, as he had very imperfect light—it being night—to cut on the outer side of the tumor, and make a more careful examination in the morning. The next day he had no difficulty in coming to the conclusion that the tumor contained the intestines of the child, and immediately attempted their replacement by manipulation. Half an hour's trial satisfied him that he could not succeed in this way, and he then concluded to open the sac. He made an incision three inches long, and the intestines came rolling out so fast that he soon had both his hands filled with them. Every inch of the small intestines had been confined in the tumor, and from its construction and the presence of the patch of natural skin, Dr. Beaton had no doubt they formed and matured there. The process of returning or transmitting them to the abdomen was necessarily slow, as the opening was very small, and they were considerably distended with gas. The inconvenience of the presence of gas became greater as the work proceeded, and at length he had to resort to pricking the bowel, in order to allow it to escape. The pricking was continued until the whole had been returned. The cord was then tied at the proper place, the abnormal appendage cut off, a pad adjusted, and the child dressed. A teaspoonful of castor-oil was ordered, and on his return, four hours afterwards, he learned that it had "operated nicely." The child thrived as well as any child could, and is now a fine healthy little fellow.

